# Torsional Crack Localization in Palm Oil Clinker Concrete Using Acoustic Emission Method

**DOI:** 10.3390/ma14185446

**Published:** 2021-09-20

**Authors:** Safdar Khan, Soon Poh Yap, Chee Ghuan Tan, Reventheran Ganasan, Muhammad M. Sherif, Ahmed El-Shafie

**Affiliations:** 1 Department of Civil Engineering, Faculty of Engineering, Universiti Malaya, Kuala Lumpur 50603, Malaysia; safdarkhan12ce12@gmail.com (S.K.); spyap@um.edu.my (S.P.Y.); reven0207@gmail.com (R.G.); elshafie@um.edu.my (A.E.-S.); 2 Department of Civil, Construction and Environmental Engineering, School of Engineering, University of Alabama at Birmingham, Birmingham, AL 35294, USA; msherif@uab.edu

**Keywords:** mechanical properties, palm oil clinker, torsional behavior, acoustic emission

## Abstract

Palm oil clinker (POC) aggregates is a viable alternative to the naturally occurring sand and gravel in the manufacturing of concrete. The usage of POC aggregates assists in the reduction of solid waste and preserves the consumption of natural resources. Although researchers investigated the mechanical response of POC-containing concrete, limited research is available for its torsional behavior. In general, the torsional strength depends on the tensile strength of concrete. This research investigates the compressive, tensile, and torsional response of concrete with various ratios of POC-aggregates. Five batches of concrete were casted with POC-aggregate replacing granite at ratios of 0, 20, 40, 60, and 100%. The selection for the mixture proportions for the various batches was based on the design of experiments (DOE) methodology. The hard density, compressive strength, splitting tensile strength, and flexural strength of concrete with a 100% replacement of granite with POC-aggregates reduced by 8.80, 37.25, 30.94, and 14.31%, respectively. Furthermore, a reduction in initial and ultimate torque was observed. While cracks increased with the increase in POC-aggregates. Finally, the cracking of concrete subjected to torsional loads was monitored and characterized by acoustic emissions (AE). The results illustrate a sudden rise in AE activities during the initiation of cracks and as the ultimate cracks were developed. This was accompanied by a sudden drop in the torque/twist curve.

## 1. Introduction

In recent years, there has been an increasing interest in recycling materials to reduce waste generated by the rapid growth in industrial sectors. Researchers have investigated the recycling of waste products to enhance the efforts of sustainable development [1]. In the construction industry, waste products could be used as an alternative to normal-weight aggregate in concrete [2,3,4,5]. The usage of waste material in concrete can lead to a reduction in weight [6,7,8,9], solid waste [10,11,12,13], CO_2_ emissions [14,15] and production cost, while preserving natural resources.

**Figure 1:**
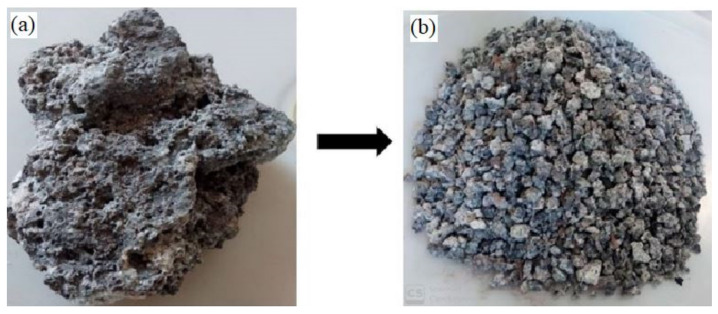
(**a**) Chunk and (**b**) grounded form of palm oil clinker.

In tropical countries, agricultural waste in the production of cementitious composites is a viable alternative [9,16]. As an example, Malaysia is the second-largest palm oil producer in the world. Large amounts of agricultural solid waste containing palm oil clinker (POC) are generated during the extraction process of palm oil. In general, POC has a low commercial value and is dumped in landfills [8,17]. Therefore, the repurposing of POC to manufacture sustainable concrete can be a viable alternative. POC is a porous material with large voids, broken edges, and irregular shapes, as seen in Figure 1a. POC can be used as an aggregate to replace conventional crushed granite in concrete [2,6,18]. POC is crushed to the required size and added to the mixture to produce POC concrete (POCC) [3].

Researchers investigated the effect of replacing the natural coarse aggregate with POC at various ratios on the compressive, tensile, and flexural strength of concrete. Results indicate that full replacement natural coarse aggregate with POC results in a reduction in compressive strength, splitting tensile strength, flexural strength, and modulus of elasticity of POCC by 40, 32, 39, and 26%, respectively [6]. However, the strength-to-density ratio of POC-based high-strength concrete is in the range of 27–31 × 10^−3^ MPa/kg·m^−3^, representing an increase of 63% when compared to normal weight concrete (NWC) [7]. The high strength-to-density ratio of POCC is due to the low bulk density of POC as compared to normal weight aggregate. Table 1 indicates that the bulk density of POC is 599.9 kg/m^3^, while granite has a bulk density of 1431.7 kg/m^3^. This could increase cost savings and provide flexibility in structural design. Ahmmad et al. [19] illustrated an enhancement of concrete performance by replacing ordinary Portland cement with fine powder of POC at a 15% ratio. It is worth mentioning, that most research on POCC has been limited to investigating the mechanical properties and long-term behavior [2,3,6,7,8,14,17,19,20,21,22].

Limited research has investigated the torsional behavior of lightweight concrete [23,24,25]. In general, POCC is a lightweight concrete, which has low tensile and torsional strengths compared to NWC. It is essential to understand that the torsional properties of POCC as torsion-loaded members can experience torsional cracks before or in combination with shear/flexural failure [26]. Thus, it is vital to study the torsional failure of the torsional-loaded concrete member as torsional failure sometimes precedes flexural failure [26]. However, the torsional behavior of POCC has not been investigated to date and to the best of the knowledge of the authors.

The evaluation of the formation and growth of cracks is essential to predict the structural integrity and performance of the POCC. The location of cracks, fractures, and delamination can impact the condition of the structure. The acoustic emission (AE) method is a nondestructive evaluation (NDE) technique that can detect and assess fractures by capturing the energy released during the formation and growth of cracks [27]. AE is noninvasive, passive, and nondestructive for the detection of crack initiation and propagation, materials degradation and corrosion, and plastic deformation. Researchers have used the noninvasive, and real-time AE methodology for investigating the fracture process in concrete [27,28,29,30,31,32]. Results indicate that the AE can localize cracks, evaluate the integrity of a structure, and provide information about a material’s responses to the applied stress [33]. The successful prediction of the behavior of torsional cracks using AE can assist in preventing torsion failure and in reducing the maintenance/repair cost of POCC and lightweight concretes.

This research investigates the use of AE methodology to detect the initiation, propagation, and localization of initial and ultimate torsional cracks in POCC subjected to torsion. Two AE parameters including the absolute energy and cumulative signal strength (CSS) were analyzed in reference to the torque/twist response. The analyses focused on the detection and identification of the damage process during crack formation, propagation of the crack, and ultimate crack in the concrete specimens. Furthermore, the AE signals were used for mapping cracks. The captured cracking profile was compared to the results of visual inspection of the POCC specimens.

## 2. Experimental Works

### 2.1. Materials and Mix Design

The materials used included cement, water, sand as fine aggregate, and granite and palm oil clinker as coarse aggregates. The cement used for all concrete batches was ordinary Portland cement (OPC) (Tasek Corporation Berhad, Ipoh, Perak, Malaysia) [34] type 1—42.5 grade with Blaine specific surface area, and specific gravity was 3510 cm^2^/g and 3.14 g/cm^3^, respectively.

Potable tap water with a pH value of 6.20 was used for the concrete mixture and for curing the concrete specimens. Two types of aggregates, crushed granite and palm oil clinker, were used as coarse aggregates. Crushed granite (Kajang, Selangor, Malaysia) was locally procured from the crushing plant and had a size in the rage of 4.75–20 mm. The POC coarse aggregate was the byproduct of the palm oil industry (Dengkil, Selangor, Malaysia) and was crushed and sieved to the required size (i.e., 4.75–14 mm) as illustrated in Figure 1a,b, respectively. The physical properties (i.e., specific gravity, water absorption [35], bulk density [36], and impact value [37]) of POC coarse aggregate were analyzed and are reported in Table 1. River sand (Kajang, Selangor, Malaysia) was used as fine aggregate in this research. Microstructural analysis was performed with Phenom Pro-X Desktop scanning electron microscopy (SEM) (Thermo Fisher Scientific, Shah Alam, Selangor, Malaysia) with an accelerating voltage of 10 kV. The surface morphology of the POC and granite particles was analyzed at 500× and 1000× magnification.

A total of six batches were casted with various proportions of POC coarse aggregate and crushed granite. The control batch (POC0) had 100% granite as coarse aggregate. The five remaining batches had 20, 40, 60, 80, and 100% of the coarse aggregate as POC. For identification, the batches are identified by POC0, POC20, POC40, POC60, POC80, and POC100. Table 2 presents the detailed mix design for each batch.

### 2.2. Specimen Preparation and Test Setup

Initially, POC coarse aggregates were 100% saturated (i.e., voids are filled with water) by soaking the aggregate in water for 24 h. This is essential to ensure the proper water-to-cement ratio is maintained as POC is highly porous when compared to granite. After soaking, the POC coarse aggregate was air-dried to attain the saturated surface dried (SSD) condition. The design of experiment methodology was implemented for ensuring that the control batch is consistent with grade 40 MPa concrete and a slump between 60 and 180 mm. For POCC specimens, the SSD POC coarse aggregate, crushed granite, and river sand were dry-mixed for 2 min to obtain a homogenous mass. The OPC and 70% of water were added and mixed for another 5 min. Finally, the remaining water was added to obtain the required slump value. Three layers of the fresh concrete was casted in the molds, and a vibrating table (Allam Vibrating Table, Seremban, Negeri Sembilan, Malaysia) was used for the compaction. After 24 h, the specimens were extracted from the molds, and the specimens were cured in water for 28 days and then tested.

Cubes of 100 mm were fabricated to investigate the compressive strength, water absorption, and ultrasonic pulse velocity (UPV) [38,39,40]. Three specimens were prepared and tested at 28 days of curing for each batch. In addition, cylindrical specimens and prisms with dimensions of 200 mm in height by 100 mm in diameter, and 500 × 100 × 100 mm^3^ were casted for investigating the splitting tensile strength and flexural strength of the specimens, respectively [41,42].

**Figure 2:**
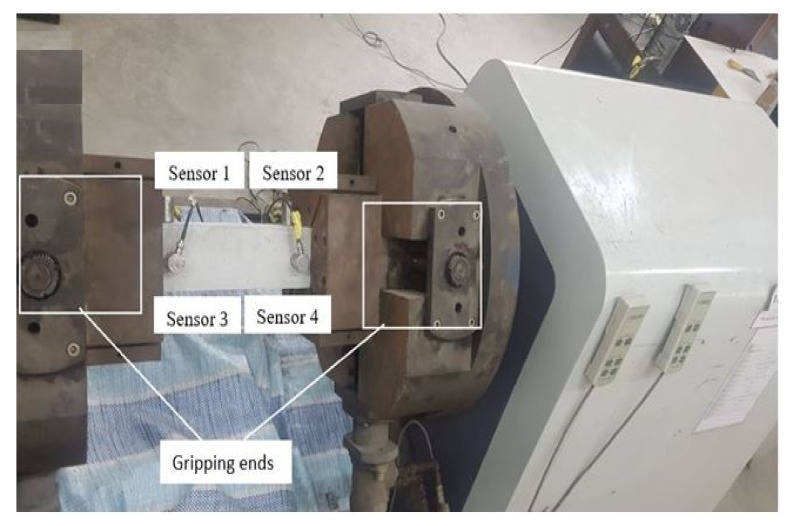
Torsion machine and acoustic emission method.

**Figure 3:**
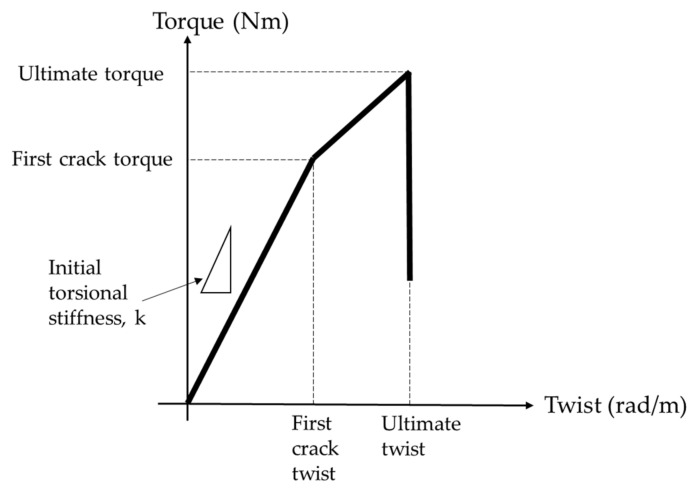
Modified torsional model up to ultimate cracks.

Furthermore, three prism specimens with dimensions of 500 × 100 × 100 mm^3^ were casted to investigate the torsional response. An automated torsion machine (GT Instruments Sdn Bhd, Puchong, Selangor, Malaysia) shown in Figure 2 was used to apply a constant torsional strain rate of 0.15 degrees/min. The acoustic emission methodology was used to detect the initial and ultimate crack of the concrete. The torsional response was measured directly from the torsion machine. The initial torsional stiffness and initial and ultimate toughness were calculated based on the Figure 3, which was modified from the simplified torsional model from [43]. The initial stiffness was calculated as the linear gradient before the formation of the first crack. In addition, the initial and ultimate toughness were calculated as the total area under the initial and the ultimate torque/twist responses.

To identify the cracking behavior of the specimens subjected to torsional load, four AE sensors with a resonant frequency of 400 kHz were mounted on the specimens. The sensors were attached using electron wax coupling agent at the two-perpendicular sides with a separation distance of 230 mm, as shown in Figure 2. To enhance the signal, 40 dB preamplifiers were used. A six-channels PCI-2 AE System (MITRAS Group, Inc.) manufactured by Physical Acoustics Corporation (PAC, MISTRAS Group, Princeton, NJ, USA) was employed. Several researchers have incorporated this system to accurately determine the cracking behavior of concrete specimens. The data were analyzed using the acoustic emission software (AE win, Version E5.30, 2013, PAC, MISTRAS Group, Princeton, NJ, USA) with an emphasis on the absolute energy, CSS, and AE amplitude. The AE sampling rate was set at 2 MHz, with the pretrigger of 256 μs and data length at 2.024 ms. The peak definition time (PDT), hit definition time (HDT), and hit lockout time (HLT) were configured as 50, 150, and 300 μs, respectively. The pass filters for all channels were set between 20 and 400 kHz. In addition, the AE threshold was set at 40 dB. A pencil lead fracture (PLF) test was conducted for each sensor to check the sensitivity and coupling properties of the sensors to the specimen. Finally, the location and pattern of cracks of the specimens were mapped using the AE results and compared to observations during visual inspection.

## 3. Results and Discussion

### 3.1. Mechanical Properties

**Figure 4:**
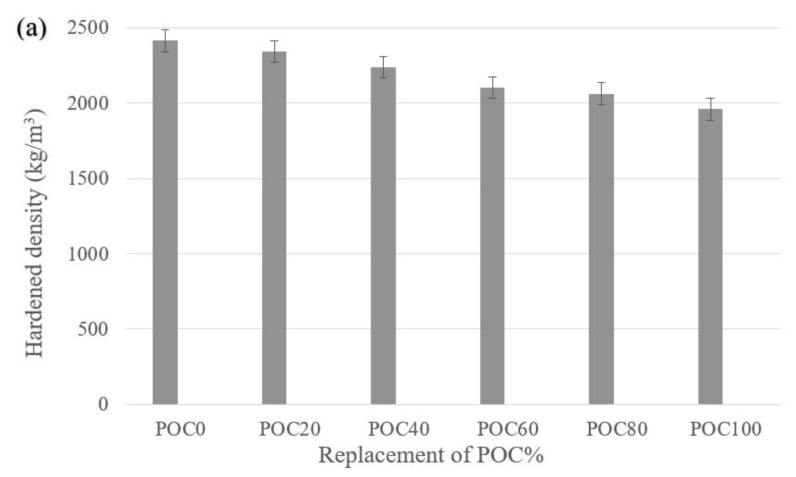

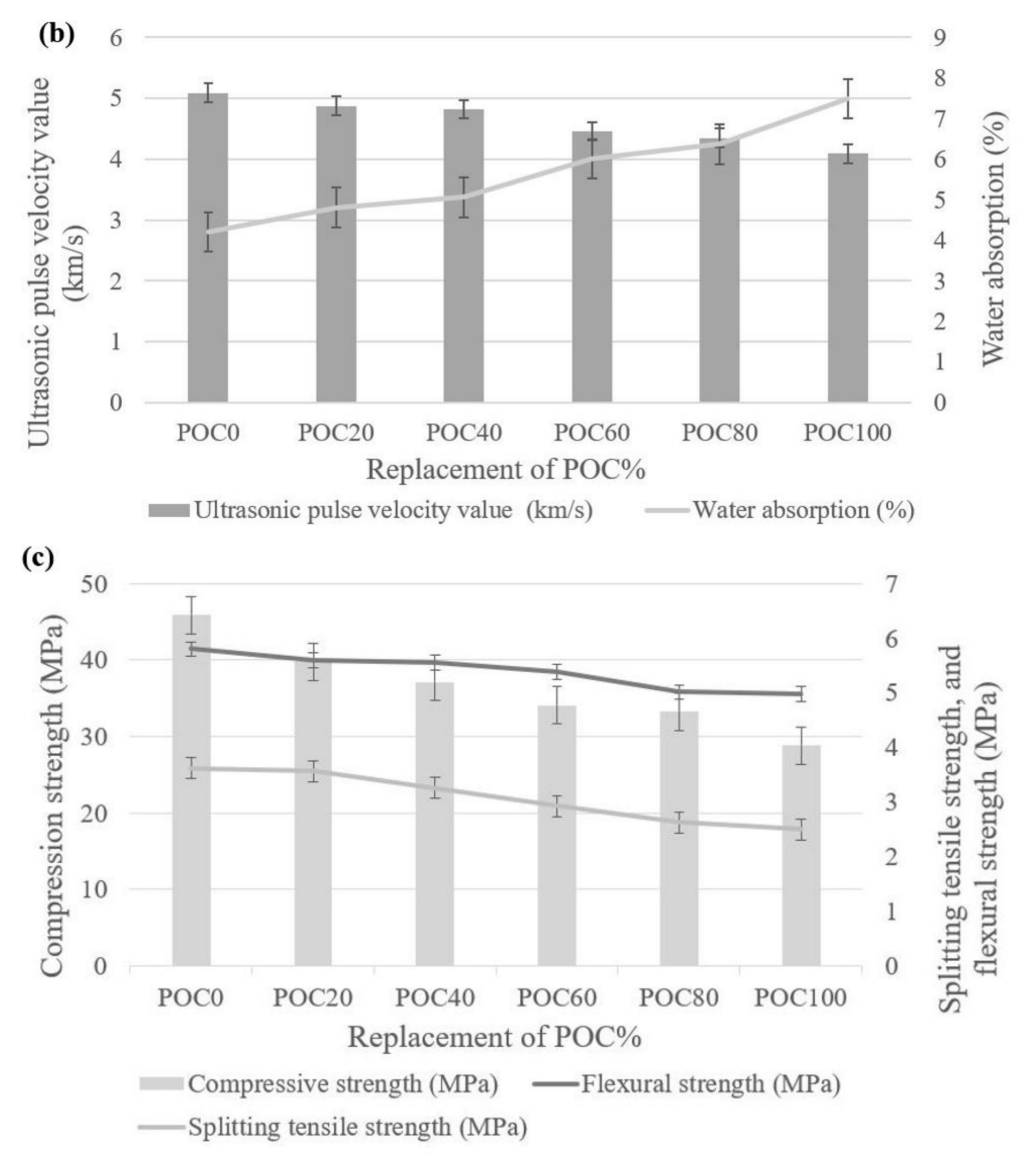
Mechanical properties of palm oil clinker concrete. (**a**) Hardened density, (**b**) ultrasonic pulse velocity value and water absorption, and (**c**) compression strength, splitting tensile strength, and flexural strength at different replacements level of POC.

Figure 4 represents the mechanical properties (i.e., hardened density, compression strength, water absorption, UPV value, splitting tensile strength, and flexural strength) of the various batches investigated. The hardened density and compressive strength of concrete decreased as the POC ratio increased. The maximum reduction in hardened density and compressive strength of 18.83 and 37.25%, respectively, was observed for specimens with 100% POC. The reduction in hardened density can be attributed to the small bulk density of the POC as compared to the granite. The decrease in strength of POCC is due to the porous surface and internal voids of POC coarse aggregates, as illustrated in Figure 1. This resulted in a reduction in load-bearing capacity. Furthermore, POC has low strength when compared to granite [2,3,44,45], as presented in Table 1.

The water absorption of concrete increased as the POC ratio increased as shown in Figure 4b. The maximum increase was observed for POC100 with a water absorption of 78% higher than that observed for the POC0 specimens. This can be attributed to the larger voids and water absorption of POC compared to granite. Lo et al. [46] indicated that aggregates with water absorption ranging between 8.9 and 11% have a void area ranging from 14.4 to 21.7%. In addition, aggregate with high water absorption produces a greater percentage of porous medium at the concrete interfacial zone.

**Figure 5:**
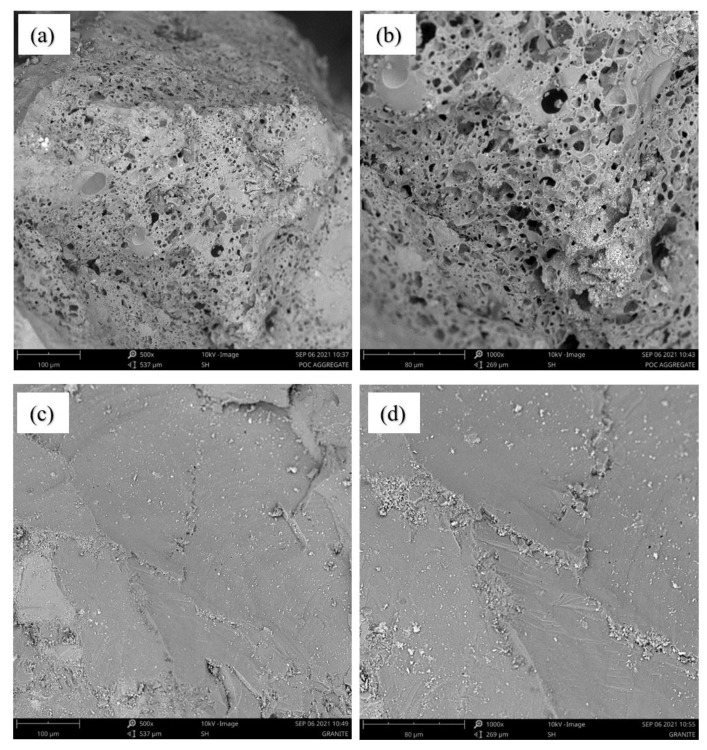
Scanning electron microscopy images for POC aggregates surface texture at (**a**) 500× and (**b**) 1000× magnifications, and granite aggregates at (**c**) 500× and (**d**) 1000× magnifications.

The UPV value of the concrete reduced with the increase in the ratio of POC coarse aggregate. The maximum reduction in the UPV value was observed at 19.64% for POC100 specimens as compared to POC0 specimens. This is due to the porous structure of the POC coarse aggregate, as shown in Figure 5a. The pulse velocity is reduced through the concrete specimen due to the impeding effect of air in empty pores between or inside the aggregates [8,47]. In general, the concrete properties are considered good when the UPV is between 3.66 and 4.58 km/s [7]. The UPV values of oil palm shell (OPS) and POC-based high-strength lightweight concrete (HSLWC) are higher than 3.66 km/s after curing by 1 day and above due to the existence of small pores and cracks. Therefore, the results obtained for the UPV value for all specimens were in the range of 4–5 km/s, as shown in Figure 4c. This indicates that the specimens had small pores and cracks.

**Figure 6:**
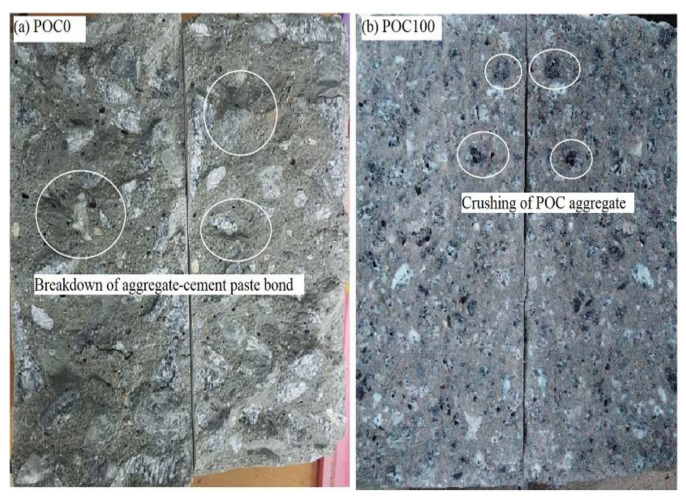
Cross-sections of the failed specimens under splitting tensile test. (**a**) POC0 and (**b**) POC100 mixes.

The splitting tensile strength was reduced with the increasing ratio of POC. A maximum reduction in splitting tensile strength of 30.94% was observed for POC100 specimens as compared to the POC0 specimens. The reduction in strength is due to the failure of the POC aggregate and at the aggregate-matrix interface [6]. This finding is enhanced by examining the cross-section at the crack interface of the failed specimen in Figure 6. Ahmad et al. [48] indicated that the 28 days splitting tensile strength of POCC was 16–32% lower than the NWC.

The flexural strength of concrete was reduced with the increasing ratio of POC. A maximum reduction of flexural strength at 14.31% was observed for POC100 specimen when compared to POC0 specimens. This can be attributed to the low aggregate crushing strength of the POC as compared to granite [6]. Aslam et al. [49] reported the 28 days flexural strength of POCC is in the range of 5–6 MPa and that the compressive strength ranged from 34 to 55 MPa. In this research, the flexural strength of all specimens except for POC100 specimens was higher than 5 MPa. This can be attributed to the specimens experiencing hardened densities greater than 2000 kg/m^3^, except for POC100 specimens as illustrated in Figure 4c.

Figure 5 displays the SEM micrographs of both the POC and granite aggregates used in this research. It can be observed from the Figure 5a,b that the POC has a higher porosity as compared to the granite as illustrated by Figure 5c,d. This results in the poor mechanical response of POCC specimens.

### 3.2. Torsional Behavior

In general, the cracking torsional stiffness of concrete is dependent on their tensile strength. The torsional failure occurs by the development of tensile stresses [24]. Table 3 summarizes the parameters of the torsional response for the various specimens investigated. The torsional parameters include: cracking torque/twist, ultimate torque/twist, initial stiffness, initial toughness, and the ultimate toughness. The cracking torque/twist corresponds to the torque/twist at crack initiation. The cracking torque of concrete decreased as the ratio of POC increased except for POC40 specimens. Among POCC specimens, POC40 had the maximum cracking torque specimens of 57.77 Nm and had the smallest cracking twist of 0.0046 rad/m. This corresponded to 4.68% lower than the control (POC0) specimens and 1.3 times greater than the cracking torque of POC100 specimens. In addition, POC40 experienced the maximum cracking torsional stiffness of 11.47 kNm^2^ when compared to all POCC specimens. The control specimens had the maximum cracking torsional stiffness due to cracking at the smallest angle of twist. A maximum reduction in cracking torque and torsional stiffness of 28.42 and 38.97%, respectively, was experienced by POC100 specimens when compared to the control (POC0) specimens. It is worth noting, that POC60 specimens sustained a high cracking torque of 47.18 Nm at the maximum twist of 0.0060 rad/m.

**Figure 7:**
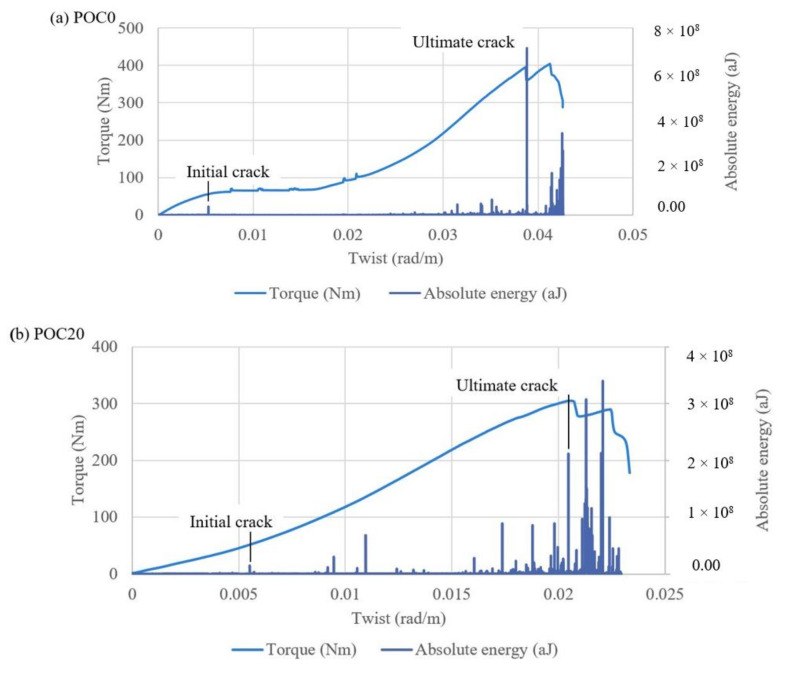

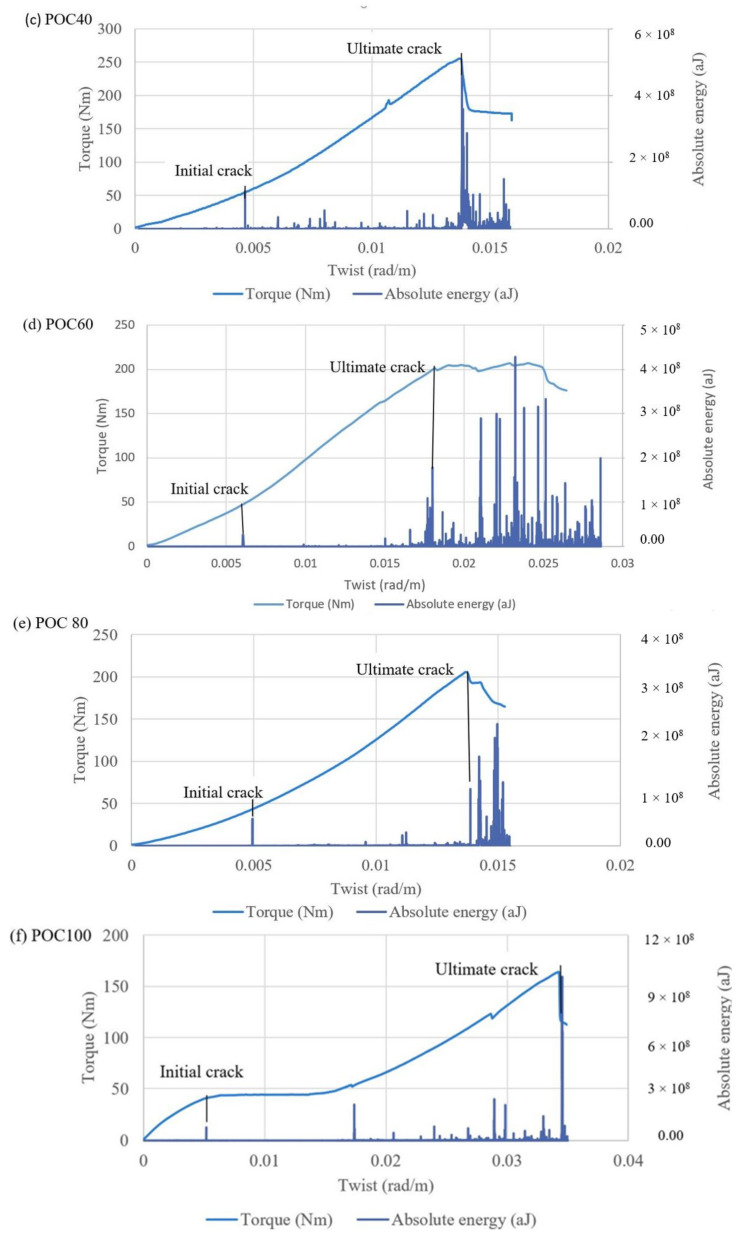
Absolute energy and torque versus angle of twist graphs for (**a**) 0%, (**b**) 20%, (**c**) 40%, (**d**) 60%, (**e**) 80%, and (**f**) 100% replacement levels of POC coarse aggregate.

The initial torsional toughness decreased with the increasing ratio of POC. The maximum reduction in initial torsional toughness of 31% occurred for POC100 specimens when compared to control (POC0) specimens. However, the twist of POC100 that resisted the cracking torque was almost 8% smaller than the twist of POC0 specimens at the cracking torque. The torsional stiffness is not dependent on the type of reinforcement of the concrete [43]. Therefore, all specimens experienced a linear torque/twist response up to the initial crack or cracking torque from the torque/twist response as seen in Figure 7.

The torsional capacity of concrete depends on the concrete strength; however, due to the absence of reinforcement, the cracks initiated suddenly and propagated rapidly [40]. The ultimate torque decreased as the POC coarse aggregate quantity increased. POC100 specimens had the least ultimate torque of 164.37 Nm at the largest twist angle of 0.034 rad/m. POC20 specimens experienced the highest torsional toughness of 2.84 Nm/m among POCC specimens, while POC100 specimens experienced the second highest torsional toughness of 2.48 Nm/m, when compared to POCC specimens. The high ultimate torsional toughness is attributed to the specimens resisting the ultimate torque at large twist angles. This resulted in an increase in the area under the torque/twist curve. It is worth noting that the torsional strength is highly dependent on the splitting tensile strength. Therefore, the torque reduced with the substitution of aggregate with POC.

The ultimate twist decreased upon increasing the POC ratio from 0 to 40%. Beyond a 40% ratio of POC, the specimens had an increase in the ultimate twist. A large ultimate twist of 0.039 and 0.034 rad/m, was observed for POC0 and POC100 specimens, respectively. This indicates that POC0 and POC100 specimens have high resistance to ultimate cracks due to homogeneity and the existence of a single type of coarse aggregate (i.e., 100% of granite or 100% of POC). However, POC100 specimens resist the lowest torque, while POC0 specimens exhibit the maximum ultimate toque. This can be attributed to the low impact and crushing strength of the POC aggregates as compared to granite.

All initial cracks or cracking torque of the mixes occurred in the range of 14–25% of the ultimate torque. The POC100 specimens had an initial crack torque of 25% of the ultimate torque. POC0 and POC100 had similar initial and ultimate twists. All specimens failed at ultimate torque and did not experience torque failure. This is due to the brittleness of concrete. Furthermore, all specimens experienced a sudden drop in the torque/twist response at ultimate cracking and the maximum fracture energy was released.

### 3.3. Acoustic Emission Method for Crack Detection

The AE methodology is extremely sensitive for the detection of crack initiation and propagation. Furthermore, it can identify and detect microcracks that are induced in at early stages [50]. Shahidan et al. [51] indicated that the two parameters of AE technique absolute energy and signal strength are suited for the classification of damage in real time for a member exposed to a loading scenario. Therefore, the parameters used in this research for the AE investigation at initial and ultimate cracking are the cumulative signal strength and absolute energy parameter analysis.

#### 3.3.1. Absolute Energy

The quantifiable measurement energy that consists of all events and AE hits is known as absolute energy [50]. The torque/twist curves were compared to the evolution of absolute energy to obtain the initial and ultimate torques and their corresponding twists. In addition, it is worth mentioning that the absolute energy history was used for investigating the cracking torque and initial cracks. After applying torque on the specimens, the absolute energy did not show any response during the uncracked stage, as no fracture energy was released. After some time, cracks were initiated. These small cracks, invisible to the naked eye, did not occur on the surface of the sample and could not be detected. Before the occurrence of the first crack, the intensity of AE activities was very low. Post the formation of the initial crack, there was a rise in the absolute energy as energy was released at a high torque.

The initial cracks from the absolute energy and torque/twist graph for the specimens are shown in Figure 7. At the initial cracking, the angles of twist for the specimens were in the range of 0.0046–0.0056 rad/m. The angle of twist at initial cracking is known as cracking or initial twist. The sudden increase in absolute energy for POC100 specimens occurring at a cracking twist was 0.0052 rad/m. The increment in the absolute energy indicates that the initial twist was 0.0060 rad/m for POC60 specimens, which is greater than the initial twist for POC40 specimens. This indicates that POC60 specimens have a high resistance to the initial cracking when compared to POC40 specimens.

In general, the absolute energy reduced after the formation of the initial cracks. Post the initial cracking of the specimens, low AE activities can be attributed to the propagation of the existing crack. In addition, microcracks were observed on the surface of the concrete specimen as the applied torque increased. When new cracks developed, a jump in the absolute energy graph was accompanied by a drop in torque. This is due to the release of high fracture energy as cracks are formed. The ultimate crack is formed at the maximum AE activities, accompanied by the maximum torque. The specimens failed at the ultimate crack, and a drop in the torque/twist curve was observed. Table 4 presents the recorded absolute energy for all batches with their corresponding twists at initial and ultimate cracks. The angle of twist during the ultimate cracking ranged from 0.014 to 0.039 rad/m. The increase in absolute energy occurred at high ultimate twist angles of 0.039 and 0.034 rad/m, for POC0 and POC100 specimens, respectively.

#### 3.3.2. Cumulative Signal Strength (CSS)

**Figure 8:**
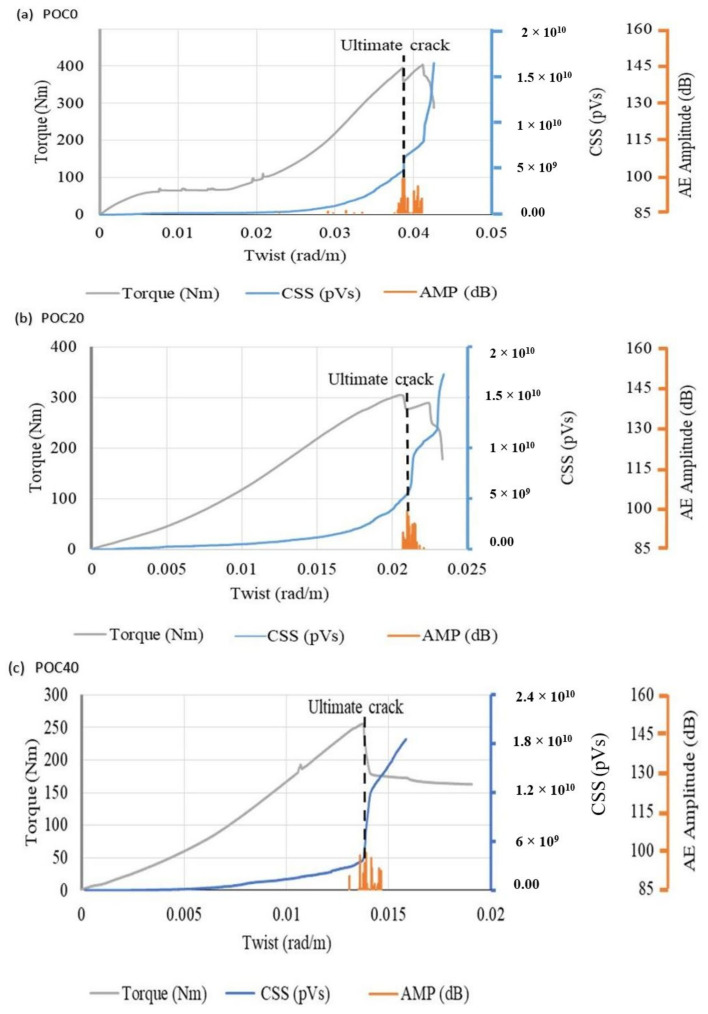

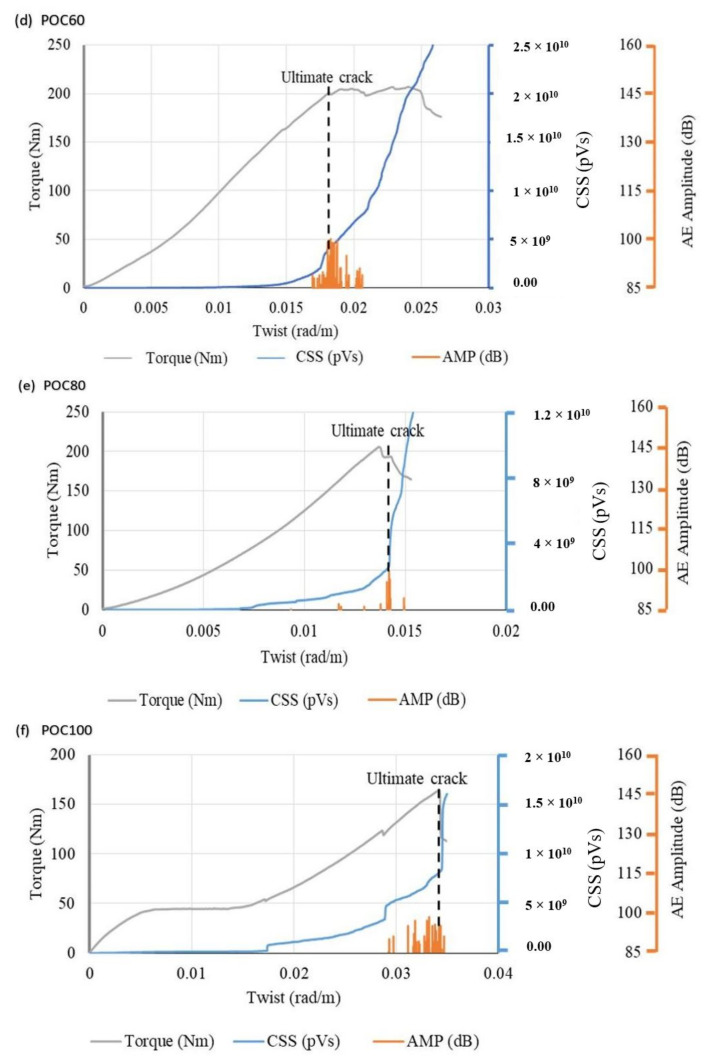
CSS, AE amplitude, and torque versus angle of twist graphs for (**a**) 0%, (**b**) 20%, (**c**) 40%, (**d**) 60%, (**e**) 80%, and (**f**) 100% replacement levels of POC coarse aggregate.

The area covered by the voltage signal of AE over the period of the waveforms is known as signal strength [28]. In addition, the cumulative summation of the signal strength is known as cumulative signal strength (CSS). It can be seen that the CSS and amplitude did not show any response to the initial crack as illustrated in Figure 8. This is due to the dependence of CSS on the number of AE events. The AE events increased with the increase in torque and resulted in an increase in the intensity of CSS graphs for all specimens after 50% of the ultimate torque was applied. The increase in intensity of CSS is attributed to the propagation of cracks and development of new cracks.

At the formation of new cracks, a hike in the CSS graph was observed. Also at the ultimate torque, a high fracture energy was released, which in turn increased the CSS. The same phenomena were seen in the signal strength-time response that increased suddenly at the ultimate crack [52]. POC0 and POC100 had high values of CSS and resisted the ultimate cracks at high ultimate twist of 0.039 and 0.034 rad/m, respectively. The increase in the intensity of the CSS graph at the low ultimate twist was recorded for POC40 as shown in Figure 8c. Thus, CSS is a better parameter for the detection of the growth of cracks and angle of twist at which ultimate torque is applied. However, the CSS is incapable of identifying the initial cracks inside the concrete specimens.

In addition, the history of the AE amplitude was investigated for all specimens as illustrated by Figure 8. The results indicate negligible response of the AE amplitude during the initial cracking stage. Furthermore, the AE amplitude is in agreement with the observations of the CSS response.

### 3.4. Cracks

**Figure 9:**
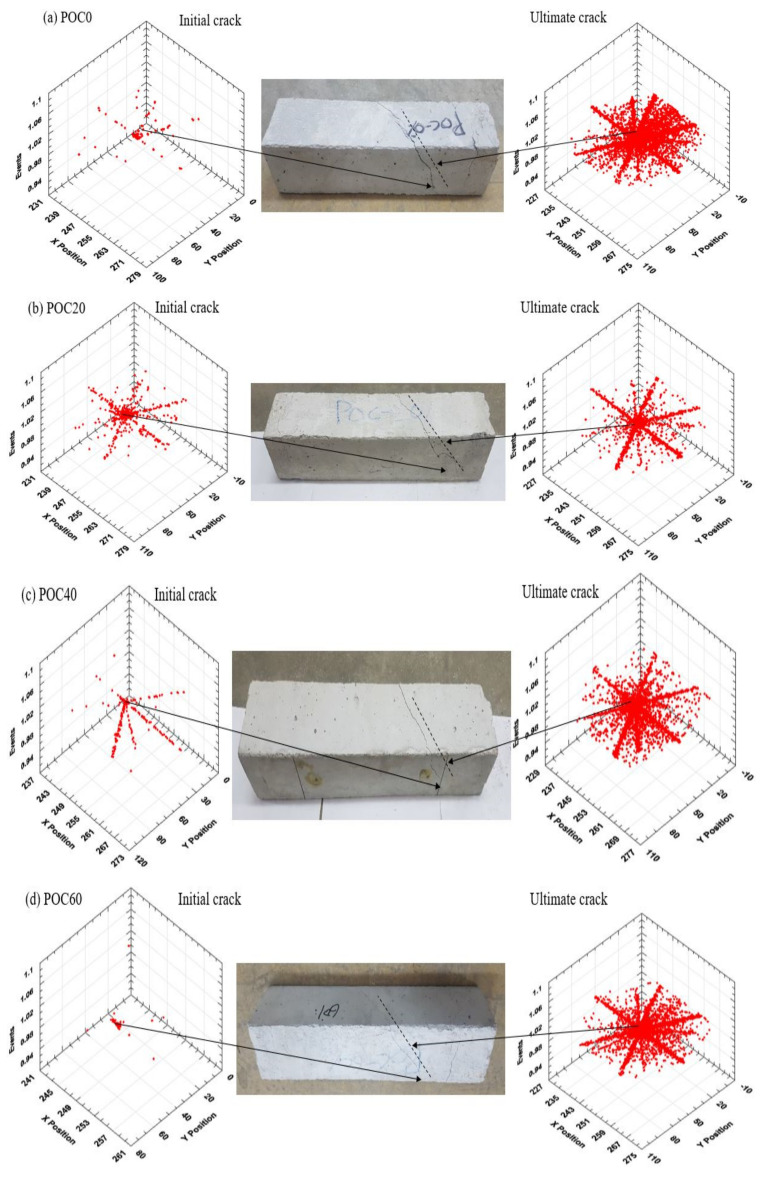

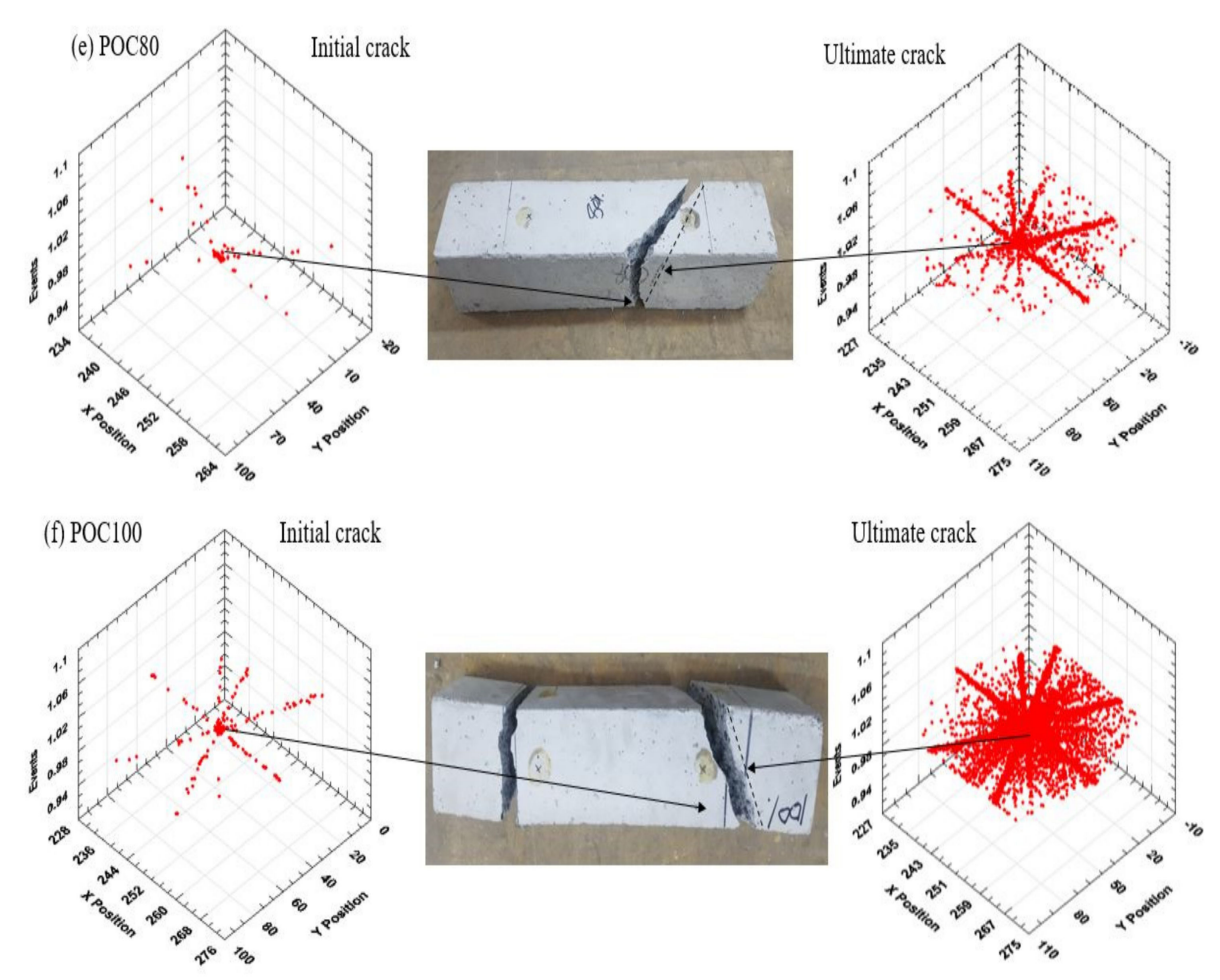
Crack mapping and profile of initial cracks, concrete specimen, and ultimate cracks for (**a**) 0%, (**b**) 20%, (**c**) 40%, (**d**) 60%, (**e**) 80%, and (**f**) 100% replacement levels of POC coarse aggregate.

Figure 9 illustrates the mapping and profiling of cracks for the various specimens at initial and ultimate torque. The results indicate that the crack width increased with the increase in POC ratio. In general, cracks initiated from the edge of the fixed end of the torsion machine and propagated on the surface towards the moveable end of the torsion machine at an inclination angle ranging between 43 and 48 degrees. The failure pattern of all specimens had a skewed bending failure. High intensities of acoustic events were recorded upon reaching the ultimate torque at which the failure of specimens occurred.

#### 3.4.1. Comparison between POC 0% and POC 100%

The initial crack of POC0 and POC100 specimens initiated from 232 mm of the x-axis from the fixed end of the machine and then spread on the y- and z-axis, as shown in Figure 9a,f. Then, the propagation of cracks was observed on the concrete specimen surface with the increase in the applied torque. The crack mapping of the initial and ultimate cracks show that the POC100 specimens broke into three pieces, while POC0 specimens had a small crack opening on the surface.

#### 3.4.2. Comparison between the Mixes

The initial crack of POC60 started at a higher position, i.e., at 242 and 21 mm on the x-axis and y-axis, respectively, as illustrated in Figure 9d. After some time of the initial crack, the cracks propagated on the surface of concrete specimens accompanied by an increase in the AE activities in all directions (Figure 9). The width of the ultimate crack up to POC60 increased gradually, and the specimen did not split into parts. The crack width of POC80 and POC100 specimens increased drastically, and the samples split into two and three parts, respectively. Low intensities of events were recorded for the POC40 and POC80, as shown in Figure 9c,e. The expansion of the ultimate cracks was approximately 258, 70, and 1 mm on x-, y-, and z-axis for all specimens, respectively.

## 4. Conclusions

The following conclusions were drawn from the analysis and discussion of the experimental results of this research

- Maximum reductions of 18.83, 37.25, 30.94, and 14.31% in hard density, compression strength, splitting tensile strength, and flexural strength of concrete, respectively, occurred when POC was fully substituted for granite in the concrete mix. Meanwhile, the water absorption of the POC100 specimens was 1.78 times higher than that of POC0 specimens.
- The initial and ultimate torque decreased by increasing the amount of POC and showed a reciprocal relationship with the initial and ultimate cracks. POC40 specimens had the maximum initial torque among all POCC specimens. In addition, POC40 specimens had high initial torsional stiffness but had the lowest angle of twist when compared with all specimens.
- POC100 specimens sustained the initial and ultimate torque at a large angle of twist and was comparable to the control (POC0) specimens. However, the initial torsional stiffness and ultimate torsional toughness of POC100 were 1.64 and 2 times smaller than the control (POC0) specimens, respectively.
- The absolute energy was found to be a useful AE parameter for the detection of both initial and ultimate torsional cracks. On the other hand, the AE amplitude and CSS parameter were found to be capable of identifying the growth of cracks and the ultimate torsional cracks. High-intensity AE events were recorded, and high-energy dissipations were observed for POC0 and POC100 specimens. The initial crack for the tested specimens occurred almost in the range of 14–25% of the ultimate crack.
- The failure pattern of all tested specimens had a skewed bending failure. In addition, the width of the crack increased with the increase in the replacement ratio of granite with POC. POC80 and POC100 specimens broke into two and three pieces, respectively.

## 5. Recommendations

Further work can be carried out to study the mechanisms of torsional crack initiation and propagation, supported by microstructural analyses, which will greatly contribute to the understanding of the torsional resistance of POC concrete and other lightweight concretes.

## Figures and Tables

**Table 1:** Physical characteristics of river sand, granite, and palm oil clinker.

Properties	Aggregates		
	River Sand	Coarse Aggregates	
		Granite	POC
Aggregate size (mm)	<4.75	4.75–20	4.75–14
Specific gravity (SSD)	2.5	2.6	1.54
Specific gravity (oven dry)	2.4	2.59	1.4
Specific gravity(apparent)	2.64	2.62	1.61
Water absorption (%)	4.12	0.4	9.4
Aggregate impact value (%)	-	20.5	47.9
Bulk density (loose condition), kg/m^3^	1571.5	1431.7	599.9
Bulk density (compacted condition), kg/m^3^	1706.9	1572.1	694.4

**Table 2:** Mix proportions.

Replacement Level (%)	Batch	Water Cement Ratio	Mix Proportion (kg/m^3^)				
			Water	Ordinary Portland Cement OPC	River Sand	Granite	POC Coarse Aggregate
0	POC0	0.53	205	410	830	935	0
20	POC20	0.53	205	410	830	748	110.76
40	POC40	0.53	205	410	830	561	221.52
60	POC60	0.53	205	410	830	374	332.29
80	POC80	0.53	205	410	830	187	443.05
100	POC100	0.53	205	410	830	0	553.81

**Table 3:** Torsional behavior of concrete at different replacement level of POC.

Mix ID	Cracking Torque		Ultimate Torque		Initial Stiffness (kNm^2^)	Initial Toughness (Nm/m)	Ultimate Toughness (Nm/m)
	Torque (Nm)	Twist (rad/m)	Torque (Nm)	Twist (rad/m)			
POC0	57.46	0.0056	397.24	0.039	12.47	0.16	5.37
POC20	51.62	0.0055	305.09	0.020	9.02	0.15	2.84
POC40	54.77	0.0046	255.65	0.017	11.47	0.13	1.48
POC60	47.18	0.0060	201.20	0.018	7.81	0.13	1.64
POC80	42.61	0.0049	205.89	0.014	8.59	0.11	1.14
POC100	41.13	0.0052	164.37	0.034	7.61	0.11	2.48

**Table 4:** Initial and ultimate cracks of normal weight concrete and palm oil clinker concrete.

Mix ID	Initial Crack			Ultimate Crack		
	Absolute Energy (aJ)	Cumulative Signal Strength (pVs)	Cracking Twist (rad/m)	Absolute Energy (aJ)	CSS (pVs)	Ultimate Twist (rad/m)
POC0	3.63 × 10^7^	1.03 × 10^8^	0.0056	7.11 × 10^8^	5.44 × 10^9^	0.039
POC20	1.48 × 10^7^	2.79 × 10^8^	0.0055	2.12 × 10^8^	4.71 × 10^9^	0.020
POC40	1.12 × 10^8^	1.13 × 10^8^	0.0046	4.80 × 10^8^	4.69 × 10^9^	0.017
POC60	2.71 × 10^7^	1.47 × 10^7^	0.0060	1.79 × 10^8^	3.90 × 10^9^	0.018
POC80	5.12 × 10^7^	1.74 × 10^7^	0.0049	1.08 × 10^8^	2.26 × 10^9^	0.014
POC100	7.71 × 10^7^	8.27 × 10^7^	0.0052	9.49 × 10^8^	1.26 × 10^10^	0.034

## Data Availability

The data will be available upon request from the corresponding authors.
